# Mapping vulnerability to climate change and its repercussions on human health in Pakistan

**DOI:** 10.1186/1744-8603-8-31

**Published:** 2012-09-03

**Authors:** Sadia Mariam Malik, Haroon Awan, Niazullah Khan

**Affiliations:** 1 Department of Economics, Faculty of Liberal Arts and Professional Studies, York University, 4700 Keele Street, Toronto, ON, M3J 1P3, Canada; 2 Avicenna Consulting, 18-B, Kaghan Road, F-8/4, Islamabad, 44000, Pakistan; 3 Saiber Foundation, 18-B, Kaghan Road, F-8/4, Islamabad, 44000, Pakistan

**Keywords:** Pakistan, Climate change, Vulnerability, Health, Pakistan

## Abstract

**Background:**

Pakistan is highly vulnerable to climate change due to its geographic location, high dependence on agriculture and water resources, low adaptive capacity of its people, and weak system of emergency preparedness. This paper is the first ever attempt to rank the agro-ecological zones in Pakistan according to their vulnerability to climate change and to identify the potential health repercussions of each manifestation of climate change in the context of Pakistan.

**Methods:**

A climate change vulnerability index is constructed as an un-weighted average of three sub-indices measuring (a) the ecological exposure of each region to climate change, (b) sensitivity of the population to climate change and (c) the adaptive capacity of the population inhabiting a particular region. The regions are ranked according to the value of this index and its components. Since health is one of the most important dimensions of human wellbeing, this paper also identifies the potential health repercussions of each manifestations of climate change and links it with the key manifestations of climate change in the context of Pakistan.

**Results:**

The results indicate that Balochistan is the most vulnerable region with high sensitivity and low adaptive capacity followed by low-intensity Punjab (mostly consisting of South Punjab) and Cotton/Wheat Sindh. The health risks that each of these regions face depend upon the type of threat that they face from climate change. Greater incidence of flooding, which may occur due to climate variability, poses the risk of diarrhoea and gastroenteritis; skin and eye Infections; acute respiratory infections; and malaria. Exposure to drought poses the potential health risks in the form of food insecurity and malnutrition; anaemia; night blindness; and scurvy. Increases in temperature pose health risks of heat stroke; malaria; dengue; respiratory diseases; and cardiovascular diseases.

**Conclusion:**

The study concludes that geographical zones that are more exposed to climate change in ecological and geographic terms- such as Balochistan, Low-Intensity Punjab, and Cotton-Wheat Sindh -also happen to be the most deprived regions in Pakistan in terms of socio-economic indicators, suggesting that the government needs to direct its efforts to the socio-economic uplift of these lagging regions to reduce their vulnerability to the adverse effects of climate change.

## Background

The phenomenon of climate change – a direct consequence of an increase in atmospheric CO2 - is no longer a matter of scientific speculation but is fast becoming a reality. The increase in Earth’s surface temperature is taking place much more rapidly today than earlier in time period. The Intergovernmental Panel on Climate Change (IPCC) indicates that the average surface temperature has risen by around 0.6°C since the industrial revolution of mid 19^th^ century and is predicted to rise further by 1.1^0^C to 6.4^0^C over the 21^st^ century.^a^ According to the IPCC, 11 of the last twelve years between 1995 and 2006 have been the warmest years in the instrumental record of the Earth’s surface temperature with heat waves becoming much more frequent. Greenland, West Antarctic and Himalayan glaciers are receding fast, disrupting the supply of water, whereas the incidence of extreme weather events is becoming much more frequent.

The impact of climate change on human wellbeing is not uniform across the globe and varies according to the geographic location; ecological conditions; and the level of economic development of each country which determines the capacity of ordinary people to cope with adverse consequences. Low income countries with rampant poverty and poor living conditions suffer more than countries where living conditions of ordinary people are better and governments have the required resources to increase awareness and implement appropriate policies in an efficient manner.

Pakistan is a developing country^b^ with poor human development indicators including health. It is highly vulnerable to the adverse effects of climate change as manifested in rising temperatures, increased variability of monsoon, melting of Himalayan glaciers, and an increase in the frequency and intensity of extreme weather events and natural disasters. The greater vulnerability of Pakistan to climate change is based upon many important factors. *First*, it is a country that is highly dependent upon agriculture not only as a source of revenue and employment but also in terms of ensuring the availability of food. Given the fact that most agricultural land is rain-fed and the country at present is water stressed means that any climatic variation that affects the pattern of rainfall are likely to have dire consequences for agriculture and the associated parameters of food, employment and income. The World Food Programme [1] in Pakistan has already reported an increase in the number of food insecure districts between 2003 and 2009. Malnutrition, particularly among children under five years of age is a serious health issue with nearly one half of them estimated to be below their weight for age. Agriculture is the mainstay of Pakistan and the country has one of the highest ratios of irrigated croplands in the South Asian region with four-fifths of its total crop land being currently irrigated [2]. According to International Food Policy Research Institute (IFPRI) [3], climate change will have a major impact on three of the world’s main staple food: rice, wheat and maize from 2010 to 2050. IFPRI further predicts that global warming will decrease agricultural production by 16 percent by the year 2020 with developing countries suffering much more than the developed ones. Due to low technological and scientific base of agriculture in Pakistan, there is low capacity to adapt to changes related to climate change.

*Second*, Pakistan does not have adequate monitoring systems that predict the occurrence of extreme weather events in a timely manner. The recent flooding in Pakistan - resulting in an inundation of more than a quarter of the total land area in Pakistan and affecting close to a twenty million people^c^ - is the most recent as well as the most shocking manifestation of the lack of emergency preparedness. The economic cost of this devastation was clearly huge; the health costs were equally high. Hospitals were flooded with patients suffering from various sorts of infectious diseases including acute diarrhoea as well as skin and eye infections. The incidence of Malaria in Pakistan was reported to increase tremendously in a mere four month period following the onset of floods: international aid agencies predicted an increase of around two million cases in the four-month period compared to 1.3 million in the entire year preceding the onset of floods.^d^ This distressing situation was further aggravated by the breakdown of health services and the lack of adequate and trained health personnel.

*Third*, the majority of the country’s population is deprived in socio-economic terms thereby lacking the capacity to cope with the downside risks associated with climate change. Lack of adequate health care infrastructure and social safety net further adds to their vulnerability. Women, children and elderly population especially those that are marginalised and exposed to multiple disasters are especially vulnerable to these downside risks. According to one estimate around 40 percent of the population in Pakistan, at present, is highly vulnerable to multiple disasters [2]. Recent studies show that proper planning and adaptation strategies can minimize the damage from natural disasters. Bangladesh for instance has managed to reduce drastically the number of deaths from cyclone over time due to a combination of early warning system and cyclone shelters [4]. Similarly, the sophisticated and well planned system of emergency preparedness in Australia has helped the country to keep its death toll to a minimum.

In order to plan and implement adaptation strategies, the first crucial step is to identify geographical areas on the basis of their vulnerability to climate change. Pakistan is a highly diverse country not only in terms of its geographical and climatic features but also in terms of the development of various regions and the socio-economic conditions of people inhabiting those regions. Due to these variations, the impact of climate change is not likely to be uniform across regions within Pakistan. This paper is the first ever attempt to rank the agro-ecological zones in Pakistan according to their vulnerability to climate change. This is done by constructing an index of vulnerability to climate change that takes into account both the ecological exposure of these regions to climate change as well as the socio-economic vulnerability and the coping capacity of its inhabitants. Since health is one of the most important dimensions of human wellbeing, this paper also identifies the potential health repercussions of each of the manifestation of climate change in the context of Pakistan.

## Methods

In development literature, vulnerability is defined as the risk, faced by households, of falling below the poverty line. A sudden change of circumstances such as the loss of employment, illness, accident, or any other unforeseen event may push people - especially those without any physical or financial assets - below poverty line. Vulnerability depends upon three major factors: 1) exposure to risks 2) Sensitivity to risks and 3) adaptive capacity [4]. Climate change vulnerability, in this paper, is also defined along similar lines. It refers to risks posed by climate change to the livelihoods and assets of people and it is a function of the three factors listed above: *exposure*, *sensitivity* and *adaptive capacity*.

In this section, a Climate Change Vulnerability Index is constructed for Pakistan and estimated for various agro-ecological zones of Pakistan. Following the methodology of Heltberg and Osmolovskiy [4] the index is constructed as the un-weighted average of three sub indices consisting of (a) exposure to climate change, (b) sensitivity, and (c) coping capacity.

*Exposure* depends upon long term changes in temperature and precipitation; the frequency of extreme weather events; and weather related disasters in each ecological zone. In our index, long term changes in temperature are measured by the standard deviation of temperature as well as by the range between maximum and minimum temperature. Although range and standard deviation are both measures of variability, both of them capture distinct aspects of variability: standard deviation measures the dispersion of data around the average and therefore captures the volatility in temperature whereas range is simply the difference between the maximum and minimum value and is therefore employed to measure the extent of dispersion between the extreme values. The long term changes in precipitation are measured by the standard deviation of precipitation; and the exposure to weather related disasters due to climate variability are measured by the percentage of flood prone and drought prone districts in each ecological zone.

*Sensitivity* depends upon the extent of the reliance of region’s population on natural resource base as sources of their livelihoods; the demographic structure (e.g. children and adults would be more susceptible); the current health status of the population; and the health and sanitation facilities available to the population. We measure the extent to which the region’s population relies on natural resource base as the source of their livelihoods by share of crop income in total income and irrigated land as percentage of the total cultivable land. The demographic structure of the population is gauged by the share of population below 5 and above 65 years of age. The general health status of the population is measured by the percentage of children suffering from diarrhoea and the percentage of population that is food insecure. The health and sanitation facilities available to the population are measured by the percentage of population without access to improved water source and improved toilet facility. The selection of these variables is guided both by relevance as well as the availability of data.

*Coping capacity* refers to the ability of the population to adapt to changes in the circumstances, brought about by climate change. It depends upon the socio-economic conditions of the population exposed to climate change as well as public and private institutions. We measure socio-economic conditions of the population by household consumption per capita, the employment rate as well as the literacy rate. The quality of institution, in general is measured by some governance variables such as lack of corruption, accountability, and transparency etc. However, since data on such variables is not available at sub-national level, we focus on one measurable and relevant aspect of institutions from the point of view of our study: the provision of public goods and its coverage. We make use of data on public provision of basic health needs such as immunization services and the availability of skilled health personnel.

More specifically, the sub indices are computed as follows:

(1) $$Exposure:\;\text{E}=\left[\left({\text{sdT}}_{1}+\;{\text{sdT}}_{2}+..\;{\text{sdT}}_{12}\right)/12+\left({\text{sdP}}_{1}+\;{\text{sdP}}_{2}+..\;{\text{sdP}}_{12}\right)/12+\left({\text{rT}}_{1}+\;{\text{rT}}_{2}+..\;{\text{rT}}_{12}\right)/12\;+\;{\text{F}}_{\text{p}}+{\text{D}}_{\text{p}}\right]/5$$

Where

sdT_i_ is the standard deviation of average temperature in month i.

sdP_i_ is the standard deviation of average temperature in month i.

rT_i_ is the range between maximum and minimum temperature in month i.

F_p_ is the percentage of flood prone districts in each ecological zone.

D_p_ is the percentage of drought prone (receiving a ranking of high and medium to high in drought conditions extrapolated by Water Resource Research Institute) districts in each ecological zone

(2) $$Sensitivity:\text{S}\;=\;\left[\left({\text{s}}_{1}\;+\;{\text{s}}_{2}\right)/2\;+\;\left({\text{s}}_{3}\;+\;{\text{s}}_{4}\;+\;{\text{s}}_{5}\right)/3\;+\;\left({\text{s}}_{6}\right)\;+\;\left({\text{s}}_{7}\;+\;{\text{s}}_{8}\right)/2\right]/4$$

Where:

s_1_ = Share of population below five years of age.

s_2_ = Share of population above 65 years of age.

s_3_ = Percentage of population without access to improved water source.

s_4_ = Percentage of population without access to improved toilet facility.

s_5_ = Percentage of children suffering from Diarrhoea.

s_6_ = Percentage of population that is food insecure.

s_7_ = Irrigated land as percentage of total cultivable land.

s_8_ = Share of crop income in total income

(3) $$Adaptive\;Capacity:\text{A}\;=\left[\left({\text{a}}_{1}\right)\;+\;\left({\text{a}}_{2}\right)\;+\;\left({\text{a}}_{3}\;+\;{\text{a}}_{4}\right)/2ý+\left({\text{a}}_{5}\right)\right]\;/4$$

Where:

a_1_ = Employment Rate.

a_2_ = Literacy Rate.

a_3_ = Percentage of Children aged 12–23 months immunized against major diseases.

a_4_ = Percentage of births attended by skilled birth attendants.

a_5_ = Household consumption per capita.

All variables employed in the construction of the three sub-indices are normalized beforehand by a linear transformation using the following standard formula:

X_S_ = (X-minX)/(maxX-minX).

Where minimum and maximum values are taken from within the data.

The three sub-indices computed as above are then aggregated to obtain the Climate Change Vulnerability Index. The aggregation of these three indices is done by taking simple un-weighted average of the three. Following the methodology of Heltberg and Osmolovskiy [4], this is done to avoid arbitration in determining weights and to bypass any element of subjectivity in choosing the respective weights. We opt for this method because it is the simplest and the least arbitrary method available.

Climate Change Vulnerability Index = 1/3[(Exposure + Sensitivity + (1-Coping Capacity)].

Higher degree of exposure and sensitivity leads to higher vulnerability. However, a higher degree of coping capacity depicts lower vulnerability. For this reason the sub-index of coping capacity is subtracted from 1.

### Variables and sources of data

Table 1 provides the variables used in the present study to compute each of the sub-indices and the sources of data.

**Table 1:** Variables and sources of data for the computation of climate change vulnerability index

**Sub Indices**	**Variables**	**Source**	**Year**
**Exposure**	Standard Deviation of average temperature in month i	Pakistan Meteorological Department	1951-2009
	Standard Deviation of average yearly precipitation	Pakistan Meteorological Department	1951-2009
	Range between maximum and minimum temperature in month i	Pakistan Meteorological Department	1951-2009
	Percentage of districts that are highly prone to droughts	Water Resource Research Institute	
	Percentage of flood-prone districts	Pakistan Agricultural Research Centre (PARC)	Estimated on the basis of 2010 floods
**Sensitivity**	Share of population below five years of age	Pakistan Living Standards Measurement Survey (PSLM) District Level Data	2008-09
	Share of population above 65 years of age	PSLM District Level Data	2008-09
	Percentage of population without access to improved water source	PSLM District Level Data	2008-09
	Percentage of population without access to improved toilet facility	PSLM District Level Data	2008-09
	Percentage of children suffering from Diarrhoea	PSLM 2008–09 District Level Data	2008-09
	Percentage of population that is food insecure	World Food Programme et al. (2009)	2009
	Irrigated land as percentage of total cultivable land	Pakistan Agricultural Research Council (PARC)	2009-10
	Share of crop income in total income	Agricultural Development Bank (2005)	2002
**Adaptive Capacity**	Employment Rate	PSLM District Level Data	2008-09
	Literacy Rate	PSLM District Level Data	2008-09
	Percentage of Children aged 12–23 months immunized against major diseases *(proxy for access to health services)*	PSLM District Level Data	2008-09
	Percentage of births attended by skilled birth attendants *(proxy for access to health services)*	PSLM District Level Data	2008-09
	Household consumption per capita	PSLM District Level Data	2008-09

### Agro ecological zones

The classification of Agro Ecological Zones is based upon an Asian Development Bank study on agricultural growth and rural poverty in Pakistan [5] that divides Pakistan into nine agro-ecological zones. The corresponding districts in each of the zones are also identified by the study. Meteorological data on all districts falling within each agro-climatic zone is not available as Pakistan has a limited number of meteorological stations in the country. However, we made use of the data obtained from available meteorological stations falling within each zone to make predictions. Table 2 presents the agro-ecological zones and the districts representing each of the zones. The meteorological stations available within each zone are also listed.

**Table 2:** Agro-climatic zones of Pakistan: associated districts and meteorological stations

**Agro-climatic zones**	**Districts**	**Meteorological stations**
**Rice/Wheat Punjab**	Sialkot, Gujrat, Gujranwala, Sheikhupura, Lahore, Kasur, Narowal, Mandi Bahauddin, Hafizabad	Lahore, Sialkot
**Mixed Punjab**	Sargodha, Khushab, Jhang, Faisalabad, Toba Tek Singh, Okara	Faisalabad, Sargodha
**Cotton/Wheat Punjab**	Sahiwal, Bahawalpur, Bahawalnagar, Rahimyar Khan, Multan, Vehari, Lodhran, Khanewal, Pakpatan	Multan, Bahawalpur, Bahawalnagar
**Low Intensity Punjab**	Dera Ghazi Khan, Rajanpur, Muzaffargarh, Layyah, Mianwali, Bhakkar, Dera Ismail Khan	Mianwali, Dera Ismail Khan
**Barani (Rain-Fed) Punjab**	Attock, Jhelum, Rawalpindi, Islamabad, Chakwal	Jhelum, Islamabad
**Cotton/Wheat Sindh**	Sukkur, Khairpur, Nawabshah, Hyderabad, Tharparkar, Nowshehro feroz, Ghotki, Umerkot, Mirpurkhas, Sanghar	Nawabshah, Hyderabad
**Rice/Other Sindh**	Jacobabad, Larkana, Dadu, Thatta, Badin, Shikarpur, Karachi	Jacobabad, Larkana, Badin, Karachi
**Other North-West Frontier Province (NWFP)**	All NWFP except Dera Ismail Khan	Dera Ismail Khan
**Other Balochistan**	All Balochistan	Chaghai, Gwadar, Quetta, Zhob

Source: Asian Development Bank, 2005; and Pakistan Meteorological Department.

## Results and Discussion

Before presenting the results of the Vulnerability Index, it is useful to analyze the estimated values of the three sub indices namely: Exposure; Sensitivity; and Adaptive Capacity that make up the overall vulnerability index.

### Exposure

**Figure 1:**
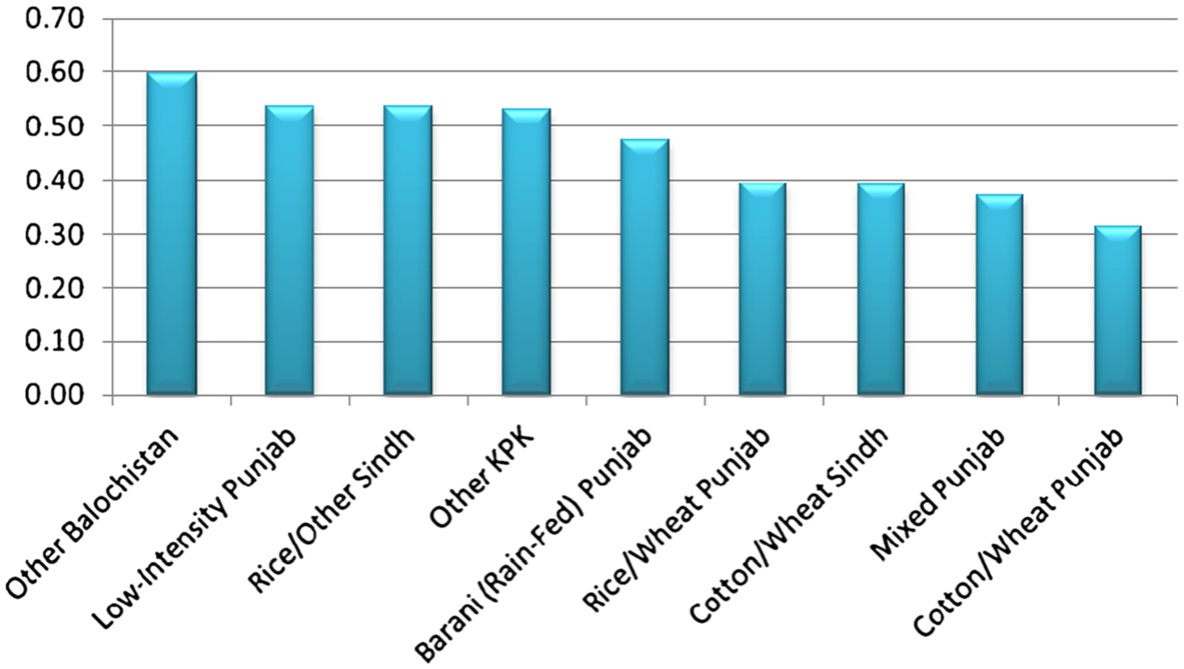
Exposure to Climate Change Index by Agro-Ecological Zones in Pakistan.

In terms of Exposure index, Balochistan region receives the highest ranking amongst all ago-ecological zones of Pakistan (refer to Figure 1). This is consistent with earlier studies conducted in the context of climate change in Pakistan. One study [6] for instance analysed changes in temperature from 1951 to 2000 by agro-ecological zones in Pakistan and found that Balochistan as well as Central and South Punjab have experienced the most pronounced changes in their mean temperature over time. The low intensity Punjab that mostly comprises of Southern Punjab receives the second place in the overall ranking.

As mentioned earlier, the exposure index is based upon five components. Table 3 provides the standardised values of these components. Notice that there is an extremely high degree of variability in precipitation in rain-fed Punjab. In terms of temperature, Balochistan depicts the highest degree of long term variability followed by Rice/Wheat Punjab. In terms of droughts, Balochistan is the most vulnerable region and in terms of flooding, KPK follwed by low-intensity Punjab and Rice-Wheat Sindh are the most vulnerable.

**Table 3:** The sub-components of climate change exposure index

**Pakistan**	**sdT**	**sdP**	**rT**	**Percentage of districts that are highly drought-prone**	**Percentage of flood prone districts**	**Exposure index (E)**
**Other Balochistan**	0.9999	0.0000	0.9786	1.00	0.00	0.60
**Low-Intensity Punjab**	0.6007	0.3475	0.9977	0.00	0.74	0.54
**Rice/Other Sindh**	0.2985	0.3060	0.1491	0.93	1.00	0.54
**Other KPK**	0.4992	0.7554	0.5199	0.00	0.88	0.53
**Barani (Rain-Fed) Punjab**	0.4408	0.8838	0.8754	0.00	0.17	0.47
**Rice/Wheat Punjab**	0.7197	1.0000	0.0000	0.00	0.25	0.39
**Cotton/Wheat Sindh**	0.0003	0.1865	0.3976	0.65	0.71	0.39
**Mixed Punjab**	0.4170	0.3071	0.7722	0.00	0.35	0.37
**Cotton/Wheat Punjab**	0.3762	0.0194	0.9174	0.00	0.25	0.31

Note: All variables are standardized.

### Sensitivity

Table 4 presents the computation results of the sensitivity index. Balochistan followed by Cotton Wheat Sindh and Low-Intensity Punjab, receives the top ranking in terms of sensitivity. Access to water and sanitation is particularly poor in Baluchistan. Cotton wheat Sindh receives top ranking in terms of share of population that is above 65 years of age. Some of the health related indicators such as the prevalance of Diarrhea is extremely high and access to toilet facilities is extremely low in Cotton-Wheat Sindh. The Low-Intensity Punjab that mostly consists of Southern Punjab has the highest proportion of population consisting of children below five years of age. Food insecurity is highest and health indicators are the poorest in this region.

**Table 4:** The sub-components of sensitivity index

**Pakistan**	**Irrigated land per capita**	**share pop < 5**	**share pop > 65**	**Unprotected water**	**Unprotected toilet**	**Diarrhoea**	**Food insecure pop.**	**Crop income share**	**Sensitivity index (S)**
**Other Balochistan**	0.08	0.66	0.37	1.00	1.00	0.17	1.00	0.48	0.63
**Cotton/Wheat Sindh**	0.06	0.00	1.00	0.25	0.97	0.91	0.58	1.00	0.58
**Low-Intensity Punjab**	0.05	1.00	0.12	0.17	0.75	1.00	0.53	0.78	0.54
**Cotton/Wheat Punjab**	0.05	0.90	0.60	0.04	0.55	0.91	0.13	0.94	0.47
**Other KPK**	0.37	0.33	0.16	0.46	0.39	0.59	0.82	0.16	0.45
**Rice/Other Sindh**	0.14	0.82	0.00	0.15	0.32	0.96	0.19	0.86	0.40
**Mixed Punjab**	0.06	0.51	0.84	0.12	0.42	0.80	0.07	0.53	0.37
**Barani (Rain-Fed) Punjab**	1.00	0.86	0.32	0.18	0.00	0.00	0.00	0.00	0.29
**Rice/Wheat Punjab**	0.00	0.61	0.63	0.00	0.08	0.36	0.05	0.58	0.28

Note: All variables are standardized.

### Adaptive capacity

In terms of lack of adaptive capacity, Balochistan and Low Intensity Punjab top the list again (see Table 5). Per capita household consumption and access to health services is the lowest in these regions. Adaptive capacity also turns out to be quite low in KPK due primarily to its low literacy and employment rate.

**Table 5:** The sub-components of adaptive capacity index

**Agro-ecological zone**	**Working**	**Literacy**	**Immunized**	**Births by skill BA**	**Per capita HH consumption**	**Adaptivecapacity (A)**	**1-A**
**Other Balochistan**	0.33	0.01	0.00	0.00	0.00	0.08	0.92
**Low-Intensity Punjab**	0.45	0.00	0.59	0.04	0.08	0.21	0.79
**Other KPK**	0.00	0.20	0.61	0.32	0.34	0.25	0.75
**Cotton/Wheat Sindh**	0.47	0.29	0.41	0.37	0.21	0.34	0.66
**Cotton/Wheat Punjab**	1.00	0.13	0.78	0.42	0.16	0.47	0.53
**Mixed Punjab**	0.60	0.43	0.84	0.52	0.48	0.55	0.45
**Rice/Other Sindh**	0.63	0.58	0.59	0.67	0.73	0.64	0.36
**Rice/Wheat Punjab**	0.54	0.80	0.94	0.77	0.92	0.78	0.22
**Barani (Rain-Fed) Punjab**	0.27	1.00	1.00	1.00	1.00	0.82	0.18

Note: All variables are standardized.

### Aggregating the components: the computation of climate change vulnerability index

The Climate Change Vulnerability Index is obtained by aggregating the indices of Exposure, Sensitivity and Adaptive Capacity. The results are presented in Table 6. Balochistan turns out to be the most vulnerable region to climate change followed by Low-intensity Punjab. Cotton-Wheat Sindh turns out to be the next most vulnerable region.

**Table 6:** The climate change vulnerability index and its components

**Agro-ecological zones**	**Exposure (E)**	**Sensitivity (S)**	**Lack of adaptive capacity (1-A)**	**Climate change vulnerability index**	**Rank**
**Other Balochistan**	0.60	0.58	0.92	0.70	1
**Low-Intensity Punjab**	0.54	0.54	0.79	0.62	2
**Cotton/Wheat Sindh**	0.53	0.63	0.66	0.61	3
**Other KPK**	0.39	0.37	0.75	0.50	4
**Cotton/Wheat Punjab**	0.31	0.47	0.53	0.44	5
**Rice/Other Sindh**	0.47	0.45	0.36	0.43	6
**Mixed Punjab**	0.37	0.40	0.45	0.41	7
**Barani (Rain-Fed) Punjab**	0.54	0.29	0.18	0.34	8
**Rice/Wheat Punjab**	0.39	0.28	0.22	0.30	9

Rain-fed Punjab and Rice/Wheat Punjab turn out to be the least vulnerable regions. It is interesting to note that the Exposure Index is quite high in Rain-fed Punjab. However, since the region ranks low both in terms of sensitivity and lack of adaptive capacity, the overall Vulnerability Index turns outs to be low. This validates the findings of Heltberg and Osmolovskiy [4] study conducted in the context of Tajikistan according to which the overall vulnerability to climate change depends primarily on socio-economic conditions and institutional development. Improvement in socio-economic conditions reduces the vulnerability to climate change by reducing sensitivity and improving the adaptive capacity of the population.

The ranking of agro-ecological zones of Pakistan with respect to their vulnerability to climate change turns out to be quite a useful tool for designing adaptation strategies. Based upon our results, it is clear that any adaptation strategy to cope with climate change in Pakistan should focus more on Balochistan; Low-intensity Punjab; Cotton-Wheat Sindh; and KPK respectively in order of preference.

#### The health repercussions of climate change in Pakistan

In this section, we spell out some of the potential effects of climate change, particularly those related to increases in temperature and the occurrence of extreme weather events, on human health. Increase in temperature and heat waves are likely to increase the risk of heat related morbidity and mortality particularly amongst the older population groups and the urban poor. According to the WHO estimate, the rise in temperature alone has caused around 140,000 excess deaths annually from 1970 to 2004.^e^ In India, 18 heat waves were reported between 1980 and 1998 with the heat wave in 1998 affecting ten states and causing 1,300 deaths [7]. In South Asia, heat waves are most likely to occur in the rural areas affecting mostly the elderly population and outdoor workers.

Increase in the incidence and intensity of heat waves are likely to increase cardiovascular and respiratory diseases particularly amongst the elderly population. Studies have reported increase in mortality and morbidity associated with these conditions in extremely hot weather [8]. Extreme heat is also predicted to increase pollen and other aeroallergen levels triggering asthma.

Rising temperatures and humidity levels are also likely to increase the transmission of vector-borne diseases such as *Malaria, Dengue Fever, Yellow Fever* and *Encephalitis*. Studies predict that an increase of 3-4°C in average temperatures may double the reproduction rate of *Dengue* virus [9]. Other vector-borne diseases including *Schistosomiasis*, *Chagas Disease*, *Sleeping Sickness* and *River Blindness* are also projected to increase with rising temperatures.

Flooding and droughts, that may occur more frequently due to climate variability, has a direct impact on human health as it increases the risk of drowning and physical injury in addition to exacerbating water-borne infectious diseases such as Diarrhoea; Malaria; Dengue; Cholera; and Gastroenteritis [9]. In Pakistan, a dramatic increase in transmittable eye infection termed as a*cute conjunctivitis* has been reported and this increase has primarily been attributed to heavy rains and floods in Pakistan.^f^ Indirect impact of flooding on health includes reduction in food intake and health care use due to loss in livelihoods, property and employment etc. Similarly droughts increase food insecurity, malnutrition and lack of safe water thereby giving rise to various infectious diseases.

Table 7 presents the degree and type of exposure of each agro-ecological zone in Pakistan and the associated potential health repercussions. The degree of exposure is ranked as low, medium and high depending upon the corresponding rank of the vulnerability index. ‘High’ indicates the rank of 1–3; ‘Medium’ indicates the rank of 4–6; and ‘Low’ indicates the rank of 7–9 in terms of the corresponding indices of exposure; sensitivity; and adaptive capacity. The health repercussions of each manifestation of climate change are also presented in Table 7. As shown in the table, the potential health consequences of increase in temperature are Heat Stroke; Dengue; Cataract Blindness; Respiratory Diseases; and Cardiovascular diseases. The health consequences of floods in the context of Pakistan include diarrhoea and gastroentritis; skin infections; eye infections; acute respiratory infections; malaria; and mental illnesses. Droughts on the other hand, increase the risk of food insecurity and malnutrition; anaemia; night blindness; and scurvy.

**Table 7:** Mapping vulnerability to climate change and its potential health repercussions in pakistan by agro-ecological zones

		**Vulnerability to climate change**			**Health repercussions**
**Agro-Ecological Zone**	*Degree of Exposure*	*Primary Manifestations of Exposure*	*Degree of Sensitivity*	*Adaptive Capacity*	
**Other Balochistan**	High	Drought; Increase in temperature	High	Low	Food insecurity and Malnutrition; Anaemia; Night blindness; Scurvy; Heat Stroke; Malaria; Dengue; Cataract Blindness**;** Respiratory Diseases; Cardiovascular diseases
**Low-Intensity Punjab**	High	Floods; Increase in temperature	High	Low	Diarrhoea and Gastroentritis; Skin Infections; Eye Infection; Acute Respiratory Infections; Malaria; Heat Stroke; Dengue; Cataract Blindness; Respiratory Diseases; Cardiovascular diseases
**Cotton/Wheat Sindh**	High	Floods; Droughts	High	Medium	Diarrhoea and Gastroentritis; Skin Infections; Eye Infection; Acute Respiratory Infections; Malaria: Food insecurity and Malnutrition; Anaemia; Night blindness; Scurvy
**Other KPK**	Medium	Floods	low	Low	Diarrhoea and Gastroentritis; Skin Infections; Eye Infection; Acute Respiratory Infections; Malaria
**Cotton/Wheat Punjab**	low	Floods; Droughts	medium	Medium	Diarrhoea and Gastroentritis; Skin Infections; Eye Infection; Acute Respiratory Infections; Malaria; Food insecurity and Malnutrition; Anaemia; Night blindness; Scurvy
**Rice/Other Sindh**	medium	Floods; Droughts	medium	High	Diarrhoea and Gastroentritis; Skin Infections; Eye Infection; Acute Respiratory Infections; Malaria; Food insecurity and Malnutrition; Anaemia; Night blindness; Scurvy
**Mixed Punjab**	medium	Increase in temperature	medium	Medium	Heat Stroke; Malaria; Dengue; Cataract Blindness**;** Respiratory Diseases; Cardiovascular diseases
**Barani (Rain-Fed) Punjab**	high	Variability in temperature and precipitation	Low	High	Food insecurity and malnutrition
**Rice/Wheat Punjab**	medium	Increase in temperature	Low	High	Heat Stroke; Malaria; Dengue; Cataract Blindness; Respiratory Diseases; Cardiovascular diseases

Note: The ranking is done on the basis of the corresponding components of the Vulnerability Index. ‘High’ indicates the rank of 1–3; ‘Medium’ indicates the rank of 4–6; and ‘Low’ indicates the rank of 7–9 in terms of the corresponding indices of exposure; sensitivity; and adaptive capacity.

## Conclusions

Pakistan is highly vulnerable to the adverse effects of climate change, particularly those resulting from rising temperatures, increased variability of monsoon, melting of Himalayan glaciers, and an increase in the frequency and intensity of extreme weather events and natural disasters. This will have significant repercussions on human health in Pakistan not only in terms of rise in the incidence of infectious diseases – that are already inflicting a substantial proportion of its population – but also in terms of shortages in food and water that are vital to maintain good health.

Evidence from other countries shows that proper planning and adaptation strategies can minimize the adverse effect of climate change. A crucial step in this direction is the identification of regions that are especially vulnerable to climate change. This paper is an attempt to rank the agro-ecological zones in Pakistan according to their vulnerability to climate change. This is done by constructing an index of vulnerability to climate change that takes into account not only the ecological exposure of these regions to climate change but also the socio-economic vulnerability and the coping capacity of its inhabitants.

The results of our study indicate that Balochistan is the most vulnerable region with high sensitivity and low adaptive capacity. The major threats posed by climate change in Balochistan include droughts and increase in mean temperature. Low-intensity Punjab (mostly consisting of South Punjab) is the next most vulnerable region. The region is prone to floods as well as rise in temperature. The region has a high degree of sensitivity and low adaptive capacity. Cotton/Wheat Sindh is the third most vulnerable region. It is vulnerable to both floods and droughts. The degree of sensitivity is high. However the coping capacity falls in the ‘medium’ rank. The Rain-fed (Barani) Punjab has a high degree of exposure to climate change due to its greater variability in precipitation. However, since the socio-economic conditions of the people and the adaptive capacity are relatively better, the overall vulnerability to climate change turns out to be low. Based upon our results, it is clear that any adaptation strategy to cope with climate change in Pakistan should focus more on Balochistan; Low-intensity Punjab; Cotton-Wheat Sindh; and KPK respectively in order of preference. The health risks that each of these regions face depend upon the type of threat they face from climate change. Exposure to floods poses the risk of Diarrhoea and Gastroentritis; Skin Infections; Eye Infections; Acute Respiratory Infections; Malaria. Exposure to drought leads to health risks in the form of food insecurity and malnutrition; Anaemia; Night blindness; and Scurvy. Increases in temperature pose health risks such as Heat Stroke; Malaria; Dengue; Respiratory Diseases; and Cardiovascular diseases.

Finally, the results of our study indicate that Pakistan’s vulnerability to climate change not only depends upon its ecological exposure but more importantly on the socio-economic conditions and adaptive capacity of its population. The scientific ranking of agro-ecological zones in Pakistan in terms of their vulnerability to climate change shows that geographical zones that are more exposed to climate change in ecological and geographic terms- such as Balochistan, Low-Intensity Punjab, and Cotton-Wheat Sindh- also happen to be the most deprived regions in terms of socio-economic indicators. It is time that the government directs its efforts to the socio-economic uplift of these lagging regions so as to reduce their vulnerability to the adverse effects of climate change. Finally and indisputably, there is a need for all countries to move towards low carbon economies. Since low-income countries like Pakistan lack resources, development assistance needs to be transferred to these countries to help them realize this goal that is ultimately beneficially for all mankind.

## End notes

^a^http://www.who.int/bulletin/volumes/85/3/06-039503/en/index.html

^b^In 2009, the GDP per capita of Pakistan is approximately $3538 in constant US dollars (2000).

^c^Newspaper estimates.

^d^http://tribune.com.pk/story/50035/malaria-may-affect-up-to-2m/

^e^http://www.who.int/mediacentre/factsheets/fs266/en/index.html

^f^http://www.dawn.com/wps/wcm/connect/dawn-content-library/dawn/the-newspaper/local/lahore/eye-infection-on-the-rise-590.

## Competing interests

The authors declare that they have no competing interests.

## Authors’ contributions

SM, HA and NK conceived the scope, purpose and design of the research project. SM and HA conducted the relevant literature review. SM collected, reviewed and analysed the meteorological data. HA and NK collected, reviewed and analysed the health data. SM led the analysis of vulnerability index. SM, HA and NK drafted the manuscript and reviewed it critically for intellectual content. All authors read and approved the final manuscript.

## Authors’ information

Sadia Mariam Malik holds a PhD in Economics (Kansas State University, USA, 2005) and is currently Assistant Professor at York University, Canada. She was formerly the President, Centre for Research on Economic and Social Transformation (CREST), Pakistan during the conduct of this study. In past, she has also held positions as Consultant, Pakistan Institute of Development Economics (PIDE) and Director Mahbub ul Haq Human Development Centre (2006–2009), Pakistan. Her research Interests include conflict, human development, poverty, institutions, health inequality, and climate change. Dr. Haroon Awan is Avicenna Consulting. He was formerly the Director of Programme Innovations and Technical Cooperation at Sightsavers. Mr. Niaz Ullah Khan is CEO of Saiber Foundation, a non-governmental organization working in the social sector in Pakistan. He was formerly Country Director of Sightsavers in Pakistan. He has a background in Agriculture Economics and is a specialist in development management.
